# Survival-Day @ Wiesbaden business school – evaluation of a short-term educational intervention to reduce work-associated health risks during nursing internships of students in health care economics

**DOI:** 10.1186/s12995-019-0251-z

**Published:** 2019-12-04

**Authors:** Reinhard Strametz, Thomas Schneider, Andreas Pitz, Matthias Raspe

**Affiliations:** 1 Wiesbaden Business School, RheinMain University of Applied Sciences, Bleichstraße 44, 65183 Wiesbaden, Germany; 2 St. Josefs-Hospital Wiesbaden GmbH, Quality Management, Beethovenstraße 20, 65189 Wiesbaden, Germany; 3 University of Applied Science Mannheim, Professor of Social Law, Straßburger Ring 45, 68229 Mannheim, Germany; 4 Department of Internal Medicine, Infectious Diseases and Respiratory Medicine, Charité – Universitätsmedizin Berlin, corporate member of Freie Universität Berlin, Humboldt-Universität zu Berlin, and Berlin Institute of Health, Charitéplatz 1, 10117 Berlin, Germany

**Keywords:** Nursing internships, Initial training, Hand hygiene, Needle stick injuries, Second victim

## Abstract

**Background:**

In 2013 RheinMain University launched its bachelor’s degree program Health Care Economics requiring each student to participate in a mandatory two-month nursing internship. A preliminary risk assessment revealed serious risks for both students and patients and had to be addressed by appropriate measures such as mandatory systematic safety training for each student.

**Methods:**

A short-term educational intervention named “Survival-Day” was designed to minimize risks related to nursing internships of students. This intervention consists of six 45-min-units with theoretical input (2 units) and hands-on training (4 units) imparting basic knowledge and skills in CPR, hand hygiene and handling of masks and protective gowns, prevention of needle stick injuries, fire protection and firefighting. Performance of CPR was assessed using computerized manikins. Acceptance, necessity and usability were assessed anonymously by standardized written questionnaires after completion of nursing internships.

**Results:**

462 students have completed the Survival-Day until January 2019. CPR performance showed acceptable adherence rates to guideline recommendations (mean 78.8%, SD ±22.6%). The majority of students performed aseptic health care activities (66%), treated patients with multi-resistant pathogens (62%) and disposed sharp instruments such as blood-contaminated needles (76%). According to students’ self-reports about these hazardous activities, less than 50% of these students received adequate safety training at nursing facilities. However, no sentinel events such as needle stick injuries or students becoming second victim have been reported.

**Conclusion:**

Our study reveals severe discrepancies between legal obligation of nursing facilities to ensure safety instructions for nursing interns and initial training as perceived by this group. Mandatory initial training before conduction of hazardous tasks was mainly covered by our short-term educational intervention (Survival-Day). Regarding responsibility for their students a preliminary safety instruction program like the Survival-Day should be considered for all educational institutions sending students to nursing internships unless mandatory and sufficient safety trainings for nursing interns can be guaranteed by nursing facilities.

## Background

Since 2013 RheinMain University of Applied Sciences has offered a bachelor’s program in Health Care Economics (BHCE). This includes a mandatory two-month nursing internship in order to gain detailed and realistic knowledge and understanding of patient care, regarded to be crucial for sustainable professional success. The RheinMain University has formal contracts with nursing facilities defining common aspects of nursing internships, which are not specific for the health care sector. Due to greatly varying standards and capacities in these nursing facilities regarding initial trainings for interns, a general contractual agreement ensuring standardized training by all nursing facilities is not realistic.

Because of this lack of adequate training nursing internships can be categorized as danger-prone activities with relevant risks for the safety of patients and students [1–8]. Furthermore, patient care is not exclusively provided by fully trained nursing staff in health care organizations often suffering from staff shortage. Therefore risks have to be assessed systematically in order to minimize them [9–13]. Based on a systematic risk assessment previously described [14], a short-term educational intervention named “Survival-Day” was created by the RheinMain University itself to minimize work-related risks for both patients and BCHE students during their two-month nursing internship.

Undoubtedly, infectious hazards belong to the most important dangers in the area of health care [15–24]. However, the present intervention did not only address these important issues, but also others including appropriate fire protection, behavior in case of fire, basic life support and prevention of needle stick injuries.

The Survival-Day was invented to close gaps in knowledge and skills in order to empower non-nursing BHCE students to safely fulfill assigned tasks during their mandatory nursing internship since we hypothesized that induction training at nursing facilities might be inadequate due to lack of time due to staff shortage. Results of acceptance, necessity and usability of this training program will be presented in this paper.

## Methods

The intervention consists of six 45-min teaching units as shown in Table 1 divided into theoretical input and hands-on training in small groups of up to 16 students per group. All units were taught by specialized staff (e.g. paramedics, physicians and fire prevention officers). Quality of in-hospital basic life support (BLS) was assessed since July 2015 to ensure sufficient hands-on performance via computerized measurement of CPR skills for a 5-min in-hospital BLS scenario. Performance of in hospital-BLS was assessed using the algorithm software of QCPR manikins of Laerdal Medical Inc., Stavanger/Norway, based on recommendations of European Resuscitation Council (ERC) Guidelines 2010 [25]. Results are presented by percentage of compliance with ERC’s BLS algorithm as calculated by QCPR software. Scores were collected in a completely anonymized fashion and used only for analysis if students agreed after informed consent.

**Table 1:** Curriculum of the Survival-Day @ Wiesbaden Business School, each unit consists of 45 min

Unit	Topic	Content	Learning Objective
1	Fire protection and fire-fighting procedures (lecture)	• Prevention of outbreaks and spreading of fire • Securing of emergency exits and escape routes • Performance of self-help measures • Support of fire-fighters	• Students know how to minimize risk for outbreaks of fire by attending to fire protection procedures
2	CPR algorithm In-hospital Basic Life Support (lecture)	• Basic principles of CPR • ERC In-hospital BLS-algorithm • Crew resource management principles to reduce the risk of insufficient support during medical emergencies	• Students know about importance of immediate response to patients with suspected cardiac arrest and possible tasks for them in case of such emergencies
3	CPR training (hands-on training) max. 16 students/group	• Instruction how to use a bag valve mask • Supervised free hands-on training with real time feedback (Laerdal QCPR Anne Classroom Mode) in teams of two students per manikin • Five minutes drill for each student to perform in hospital BLS in teams of two students per manikin	• Students show how to perform in hospital BLS to a CPR manikin for 5 min at acceptable performance levels
4	Prevention of needle stick injuries (hands-on training) max. 16 students/group	• Demonstration of blunt cannulas for preparing intravenous medication • Demonstration of sharp cannulas and drips incl. Safeguard mechanisms • Demonstration and hands-on training of each student of secure handling and appropriate disposal of sharp instruments • Measures of post-exposure prophylaxis after needle stick injuries to prevent HIV infection	• Students know about risk of needle stick injuries, risk-reducing strategies such as safety cannulas and post-exposure prophylaxis • Students show how to safely dispose needles in a sharps disposal container.
5	Firefighting procedure (hands-on training) max. 16 students/group	• Demonstration of the safe use of a fire extinguisher • Hands-on training for each student to extinguish a fire in a fire simulator	• Students show how to safely use a hand-held fire extinguisher in a fire simulator
6	Prevention of nosocomial infections (hands-on training) max. 16 students/group	• Short lecture of five moments of hand hygiene (slides provided by German Clean Hands campaign) • Demonstration of hand disinfection using disinfectant solution with UV-indicator and UV black light. • Hands-on training for each student of hand disinfection using disinfectant solution with UV-indicator and UV black light with individual instant feedback • Demonstration and hands-on training for each student of appropriate use and disposal of protective suits (gown, gloves, face mask, cap)	• Students know about strategies and equipment to reduce nosocomial transmission of multi-resistant pathogens • Students show how to disinfect their hands correctly • Students show how to put on and remove mask and protective gown before and after contact with patients having multi-resistant pathogens

*CPR* cardiopulmonary resuscitation, *ERC* European Resuscitation Council, *BLS* basic life support.

Completion of learning objectives for firefighting, prevention of needle stick injuries, handling of protective gowns and masks and hand hygiene were assessed by teaching staff until sufficient performance was achieved during the intervention. Overall acceptance and recommendation rate were assessed for evaluation of the first event in January 2015 prior to nursing internships by using the German school grade system (1 = very good to 6 = unsatisfactory). Acceptance of this short-term intervention and on-site instructions at nursing facilities were assessed. Therefore, all students who finished their nursing internship between July 2017 and September 2018 were asked to complete a standardized content-validated questionnaire also using the German school grade system.

All questionnaires were completely anonymized to ensure standards of data protection. Anonymized use of data for scientific reasons was declared in all questionnaires. Descriptive analysis was done using Microsoft Excel© 2016.

## Results

Since the initial intervention in January 2014 a total number of 462 non-nursing BHCE students have participated in this educational intervention, as shown in Table 2. No student was allowed to enter mandatory nursing internship without completion of the Wiesbaden Survival-Day.

**Table 2:** Numbers of health care economy students participating at the Survival-Day

Semester	N =	female / male
winter 2014	43	36/7
summer 2015	59	49/10
winter 2015	66	51/15
summer 2016	55	48/7
winter 2016	67	58/9
summer 2017	50	40/10
winter 2017	45	41/4
summer 2018	24	20/4
winter 2018	53	45/8
total	462	388/74

Performance rates of in-hospital BLS showed an overall acceptable ERC BLS scores (mean 78.8%, SD ±22.6%, *n* = 346) compared to similar educational interventions [26, 27]. Learning objectives for all hands-on training sites were achieved by all participating students. No injuries or critical incidents were reported during any session of this short-term intervention.

Questionnaires for assessment of acceptance, necessity and usability were obtained from 104 BHCE students (response rate 87%). Overall acceptance of the initial session in January 2015 was very good with a mean score of 1.9 (median 2) and recommendation rate of 94%. Overall acceptance remained high after 2017 with mean grade 2.3 (median 2) compared to very mixed results for initial trainings at nursing facilities with mean grade 3.5 (median 3). Remarkably 24% of all students rated initial training at nursing sites to be deficient (grade 5) or unsatisfactory (grade 6). According to students’ feedback concerning initial training at nursing facilities 54% of all students only received a rudimental initial training, while 30% reported to have had no initial training all. Detailed aspects of non-nursing students’ tasks at nursing sites as shown in Table 3 reveal severe discrepancies between assigned tasks and required initial training: The majority of BHCE students performed hazardous tasks like aseptic health care activities, treatment of patients with multi-resistant pathogens or disposal of sharp instruments. However more than 50% of these non-nursing students performing these tasks did not receive any procedure specific safety training at nursing sites but had to refer to their knowledge and skills acquired at Survival-Day.

**Table 3:** Students’ tasks in relation to initial training at nursing facilities

Did you perform this kind of activity during your internship?	N = *	no n(%)	yes n(%)	Among all students who performed this task: Did you receive a formal initial training at your nursing site?	
				yes n(%)	no n(%)
general nursing activities	104	3 (3)	101 (97)	46 (46)	55 (54)
aseptic health care activities	92	31 (34)	61 (66)	19 (31)	42 (69)
care of patients with multi-resistant pathogens	92	35 (38)	57 (62)	30 (53)	27 (47)
care of patients with highly contagious diseases	92	66 (72)	26 (28)	10 (38)	16 (62)
disposal of sharp contaminated instruments	92	32 (35)	70 (76)	18 (26)	52 (74)
detection of /assistance during medical emergency incl. CPR	92	86 (93)	8 (9)	5 (63)	3 (38)
firefighting	92	91 (99)	1 (1)	0 (0)	1 (100)

*note: 12 students only stated to have provided general nursing leaving all other questions unanswered.

As presumed by our previous risk assessment tasks with extraordinary damage potential like detection of medical emergencies and assistance during CPR (9% of all non-nursing students) as well as firefighting (one case at all) were quite rare but did occur.

Among all 402 non-nursing BHCE students successfully completing nursing internships until January 2019 no case of needle stick injury, second victim phenomenon or other serious harm of patients or students has been reported so far.

## Discussion

Health care workers are exposed to numerous potential hazards [5, 12, 28–36]. This does also apply for non-nursing students during nursing internship justifying our short-term educational intervention. Our results indicate acceptable performance levels for all learning objectives. Detection of effectiveness however is limited to self-reported outcomes of students. Since other events of internships like observed near misses in patient care are discussed during risk management lectures during the following semester, absence of severe sentinel events like needle stick injuries or second victim incidents seems credible. Some students rating training at nursing facilities as “fair” or better (German school grade 1–4) also stated that they did not receive any initial training at all. This could be most likely explained by reduced need for training due to prior professional experience (e.g. previous training as physician assistant) in some of the students but could also possibly refer to lack of situational awareness.

Since students are free to choose any nursing facility in Germany or comparable to German nursing standards, assessment of students’ perceptions can be regarded as representative and evaluation of self-effectiveness can be regarded reasonable under the given circumstances. Quantification of risks for rare events like assistance during CPR or firefighting on a ward seemed to be adequate since these rare events occurred in this comparably small group of students with expected frequencies.

It should be assumed that deficits in initial training during nursing internships will also affect students in other educational settings such as nursing internships for high-school students or future medical students. To our knowledge there is no other preventive educational intervention like “Survival-Day” in any high-school or medical school in Germany.

## Conclusion

We implemented a short-term intervention that was widely accepted and regarded more helpful by non-nursing BHCE students preparing for their tasks at nursing internship than their received initial training at nursing facilities.

One fourth of all BHCE students reported inadequate initial training at nursing facilities in general. Also more than half of all students were exposed to hazardous activities without preliminary safety training at nursing facilities supporting our primary hypothesis of inadequate initial training of nursing interns. This justifies the need of our curricular safety training Survival-Day in order ensure necessary safety training to keep non-nursing students as well as patients free from avoidable harm that otherwise could be caused.

As long as mandatory safety trainings for all non-nursing interns are not implemented at nursing facilities, educational institutions, sending students to nursing internships should implement short-term educational interventions like the Survival-Day @ Wiesbaden Business School to ensure minimum standards of occupational and patient safety during nursing internships.

## Data Availability

The dataset is set up in the German language and therefore not suitable for international use. Interested researchers are encouraged to contact the authors for the provision of the used questionnaire or data.

## Abbreviations

BHCE: Bachelor Health Care Economics (Bachelor of Science)
BLS: basic life support
CPR: cardiopulmonary resuscitation
ERC: European Resuscitation Council
